# Strain-controlled spin transport in a two-dimensional (2D) nanomagnet

**DOI:** 10.1038/s41598-023-43025-w

**Published:** 2023-10-03

**Authors:** P. Kumari, S. Rani, S. Kar, M. Venkata Kamalakar, S. J. Ray

**Affiliations:** 1 Department of Physics, Indian Institute of Technology Patna, Bihta, 801103 India; 2 Department of Physics and Astronomy, Uppsala University, Box 516, 75120 Uppsala, Sweden

**Keywords:** Condensed-matter physics, Spintronics

## Abstract

Semiconductors with controllable electronic transport coupled with magnetic behaviour, offering programmable spin arrangements present enticing potential for next generation intelligent technologies. Integrating and linking these two properties has been a long standing challenge for material researchers. Recent discoveries in two-dimensional (2D) magnet shows an ability to tune and control the electronic and magnetic phases at ambient temperature. Here, we illustrate controlled spin transport within the magnetic phase of the 2D semiconductor CrOBr and reveal a substantial connection between its magnetic order and charge carriers. First, we systematically analyse the strain-induced electronic behaviour of 2D CrOBr using density functional theory calculations. Our study demonstrates the phase transition from a magnetic semiconductor → half metal → magnetic metal in the material under strain application, creating intriguing spin-resolved conductance with 100% spin polarisation and spin-injection efficiency. Additionally, the spin-polarised current–voltage (I–V) trend displayed conductance variations with high strain-assisted tunability and a peak-to-valley ratio as well as switching efficiency. Our study reveals that CrOBr can exhibit highly anisotropic behaviour with perfect spin filtering, offering new implications for strain engineered magneto-electronic devices.

## Introduction

Magnetism in two-dimensional (2D) van der Waals (vdW) materials has sparked intense interest due to its substantial potential for spintronic applications, relevant for quantum technology. 2D spintronic devices offer distinct advantages in terms of low energy consumption^1,2^, high storage density^3,4^, and fast device operation^5^ etc. In a 2D nanocrystal, localised magnetism can be introduced through doping, defects and engineering the edge structure as observed in the case of graphene, phosphorene^6,7^ etc. However, these materials can offer superior spin transport with larger spin lifetime and correlation, which can be useful as a channel material in a field effect transistor^8^. The existence of a long-range magnetic ordering in the 2D limit has been elusive, as the number of spin waves generated at finite temperature diverges to stabilise a magnetic ground state. Despite this, intrinsic ferromagnetic ordering was experimentally discovered recently in several 2D materials like Cr_2_Ge_2_Te_6_^9^, CrI_3_^10^, Fe_3_GeTe_2_^11^, VSe_2_^12^, and FePS_3_^13^ etc. with non-zero T_c_. However, the critical temperature of most of the 2D magnets are well below room temperature, preventing practical application for room temperature spintronics. Thereby, constant search is going on to look for new class of 2D magnets with higher critical temperature or finding ways to enhance the transition temperature invasively, through application of strain, electric field etc^14–16^.

In recent years, monolayer chromium oxyhalide monolayer CrXY (X = O, S, etc.; F, Cl, Br and I) has attracted attention^17–20^ as it offers moderately high critical temperature (CrOCl ~ 148 K) and presence of ferromagnetic semiconductor phase which is of huge technological importance. Among them, monolayer CrOF and CrOCl are FM semiconductors with large spin polarization and possess perpendicular magnetic anisotropy (PMA) which can be tuned by applying strain or electric field^17,21^. Theoretical calculations reveal that T_c_ can be enhanced upto 450 K in monolayer CrOCl in the presence of combined strain and electric field^21^. Moreover, it offers interesting electronic and magnetic phase transition under such excitations, resulting in interesting phase tunability in similar systems^22–24^. Recent experimental works reveal the presence of magnetoelastic coupling^25^ and second harmonic generation^26^ in it. Motivated by this, we have explored a sister compound of this family CrOBr, which is predicted to be a ferromagnetic semiconductor in the monolayer limit^27,28^, while the transport property of monolayer CrOBr has not been studied systematically. In this work, we performed extensive first-principles based calculations to investigate the spin-polarised electronic, magnetic and transport behaviour of monolayer CrOBr systematically in the presence of various types of strains to understand the synergy between multiple effects. The strain effect results in interesting electronic and magnetic phase transition, along with the observation of negative differential resistance (NDR) phenomenon, perfect spin-filtering and spin-injection behaviour useful for strain-controlled magneto-electronics.

## Computational details

The present study employed spin-polarised density functional theory (DFT) calculations to investigate the electronic behaviour of CrOBr through strain engineering as implemented in Quantum ATK^29^. The self-consistent calculations were carried out using the Perdew–Burke–Ernzerhof (PBE) exchange-correlation functional^30^ under the generalized gradient approximation (GGA) of the spin densities n$_{\uparrow }$(**r**) and n$_{\downarrow }$(**r**) and its gradients, of the form:

1 $$\begin{aligned} E_{xc}^{GGA} [n_{\uparrow }, n_{\downarrow }] = \int \epsilon _{xc}(n_{\uparrow }({{\textbf {r}}}), n_{\downarrow }({{\textbf {r}}}), \nabla n_{\uparrow }, \nabla n_{\downarrow } ) d^{3}r \end{aligned}$$

To account for the high on-site Coulomb interaction between the localized d-electrons of Cr, the Hubbard-U correction is considered at an energy U = 7 eV^31^. In order to increase the accuracy of our calculations, the Brillouin zone is broadened out using a *k*-point sampling of 13 × 15 × 1^32^ with density mesh cut-off of 75 Hartree. A vacuum space measuring 20 Å was built along the z-direction to dissociate the interaction between neighbouring images.

The spin transport properties in a two-probe geometry were investigated using the Non-Equilibrium Green’s function (NEGF) technique combined with DFT formalism^33,34^, in which the wave functions were expanded into a double-$\zeta$ polarised basis set using a 1 × 1 × 300 *k*-point grid. The 300 points are taken along the transport direction. In order to compute the transmission coefficient T($\varepsilon$), we used the following formula,

2 $$\begin{aligned} T(\varepsilon ) = \Gamma _{L}(\varepsilon )G(\varepsilon )\Gamma _{R} G^{\dagger }(\varepsilon ) \end{aligned}$$

Here,

3 $$\begin{aligned} G(\varepsilon ) = [(\varepsilon + i\delta _{+})S - H - \Sigma _{L}(\varepsilon ) - \Sigma _{R}(\varepsilon )]^{-1} \end{aligned}$$

and $\delta _{+}$, *S* and *H* represent an infinitesimal positive number, the overlap and Hamiltonian matrices of the entire system. The $\Sigma _{L(R)}(\varepsilon )$ is the self-interaction energy for the left (right) electrodes. The broadening function of the left (right) electrode, $\Gamma _{L(R)} = \frac{1}{i}(\Sigma _{L(R)}(\varepsilon ) - (\Sigma _{L(R)}(\varepsilon ))^{\dagger })$. In addition, the transmission function was integrated to estimate the current following Landauer–Buttiker formula given by^35,36^,

4 $$\begin{aligned} I(V) = \frac{2e}{h} \int _{\mu _{L}}^{\mu _{R}} T(\varepsilon , V) [f(\varepsilon - \mu _{L}) - f(\varepsilon - \mu _{R})]d\varepsilon \end{aligned}$$

where $T(\varepsilon$, V) represents the transmission spectrum of the electrons entering at an energy $\varepsilon$ from the left to right electrode in the presence of an applied finite bias voltage V, $f(\varepsilon )$ is the Fermi–Dirac distribution function, and $\mu _{L(R)}$ is the chemical potential of the left (right) electrode.

**Figure 1:**
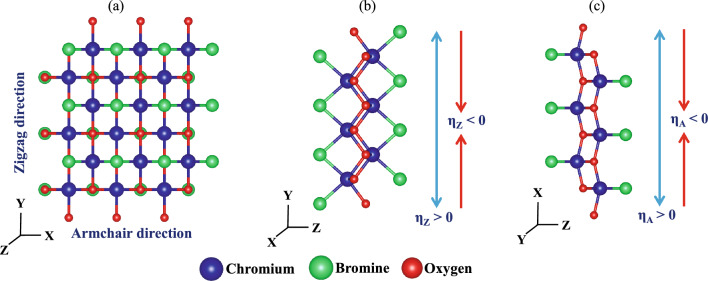
A schematic illustration of a 3 × 3 supercell of CrOBr along the (**a**) XY, (**b**) YZ, and (**c**) ZX planes. An arrow indicates the direction of applied strain in tensile and compressive cases.

## System description

CrOBr consists of a 2D network of a rectangular sublattice in an orthorhombic crystal structure (space group #59, Pmmn) in the XY plane. Its unit cell (u.c.) contains two Cr, two O, and two Br atoms with lattice parameters, a_0_ = 3.305 Å, and b_0_ = 3.882 Å . In the crystal geometry of CrOBr, O atoms surround a Cr atom and are sandwiched between Br atoms stacked parallel to the z-axis (Fig. 1). In terms of bond lengths, Cr–O and Cr–Br bonds correspond to 1.9912 Å and 2.48683 Å respectively. The bond angle in Cr–O–Cr and Br–Cr–O cases are 97.6803° and 84.9818° respectively. Throughout this study, we used a 3 × 3 sized supercell as the central unit. Here we applied both uniaxial strain as well as biaxial strain, with the former along the armchair ($\eta _{A}$) and zigzag ($\eta _{Z}$) directions of monolayer CrOBr. For each strain type, two different cases of compressive (CS) and tensile (TS) deformations are examined as shown in Fig. 1b and c, where lattice parameters have changed from pristine configuration to strained configuration of: (i) a_0_ $\rightarrow$ a, (ii) b_0_ $\rightarrow$ b. The definition of the uniaxial strain (US) along the armchair and zigzag directions are given by,

5 $$\begin{aligned} \eta _{A} = \frac{a - a_{0}}{a_{\circ }},\,\, \eta _{Z} = \frac{b - b_{0}}{b_{\circ }} \end{aligned}$$

First, we performed a complete structural relaxation of the 2D CrOBr at zero strain. This step allows the atomic positions to find their minimum energy configuration, ensuring that the material is stable and has the correct structure before applying strain. In the presence of a non-zero strain, the structure was relaxed only in the direction where no strain was applied.

**Figure 2:**
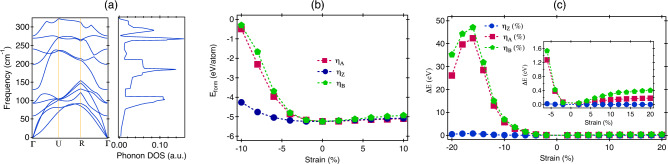
(**a**) The phonon band structure and phonon density of states of 2D-CrOBr. (**b**) The formation energy as a function of strain variable. (**c**) The variation of $\Delta$E under application of uniaxial and biaxial strain in different directions. The inset in (**c**) represents a zoomed-in version of $\Delta$E under tensile strain.

## Results and discussion

### Formation energy

An evaluation of the energetically advantageous configuration of monolayer CrOBr under strain conditions is determined by the formation energy E$_{form}$, defined as follows:

6 $$\begin{aligned} E_{form}= \frac{(E_{T} - n_{1}E_{Cr} - n_{2}E_{O} - n_{3}E_{Br})}{n_{1} + n_{2} + n_{3}} \end{aligned}$$

where, E$_{T}$ represents the total energy of the strained CrOBr, and E$_{Cr}$, E$_{O}$, E$_{Br}$ are the energy of Cr, O, and Br atoms in their bulk phase, respectively; n$_{1}$, n$_{2}$, and n$_{3}$ refer to the atom numbers in the supercell. The calculated E$_{form}$ is − 5.24 eV/atom, suggesting the possibility to synthesize monolayer CrOBr experimentally. The phonon dispersion and phonon density of the state in the unstrained configuration is shown in Fig. 2a and in Fig. S4 for different non-zero strain values^37^. The absence of negative phonon frequencies confirm the dynamical stability of 2D-CrOBr under various strain conditions. The phonon density of states indicates the presence of soft-IR phonon modes in the spectrum. The computed E$_{form}$ with strain engineering is shown in Fig. 2b and having a negative value indicates the thermodynamic stability of the system under various strain configurations. Overall, the US configurations have a lower formation energy than the BS scenario. A tiny difference in formation energy was observed under US and BS cases in the TS region, compared to the variations in the CS region where it gets enhanced rapidly with increasing the strain. The lower formation energy was observed in the presence of $\eta _{Z}$, compared to $\eta _{A}$ here. The effect of strain can be understood from the total energy difference ($\Delta$E) between the strained and unstrained system expressed by:

7 $$\begin{aligned} \Delta E = \frac{E_{s} - E_{o}}{N} \end{aligned}$$

where, $E_{s}$ is the total energy of strain configuration and E$_{o}$ is the total energy of relaxed system. The number N represents the number of atoms in the supercell. For different values of applied strain, the difference in the value of $\Delta$E between the US and BS strain cases is higher in the CS regime compared to the TS region as illustrated in Fig. 2c. $\Delta$E is found to be larger in magnitude for $\eta _{A}, \eta _{B}$ compared to $\eta _{Z}$ in the CS configuration suggesting that zigzag direction is mechanically softer than the armchair direction as it involves higher energy cost to apply strain along the armchair direction. A sudden rise in the value of $\Delta$E was observed above $\eta _{A}$ = − 6% with a monotonic enhancement between − 16% $\le \eta _{A}<$ − 6%, beyond which system looses its elasticity and can be considered as the maximum elastic limit. Similar behaviour is also observed for the case of $\eta _B$. However, no such changes were observed in the case of $\eta _Z$ and the system stays fully within the elastic limit. The increase in the CS results in the reduction of bond length between the atoms in the CrOBr layer to enhance the repulsion between them, thereby an increase in energy cost from the relaxed configuration. For TS, the dispersion between different strain branches is much smaller compared to the CS case and here also, the energy cost is higher for applying $\eta _A$ compared to $\eta _Z$. The behaviour of $\Delta$E for various strain configurations follows a similar trend to that of the formation energy behaviour. The difference in the behaviour of $\Delta$E for various strain cases indicated the presence of intrinsic mechanical anisotropy in the material. The values of bond length and bond angle in the optimized geometries under the compressive strain ($\eta _A$) are listed in Table S1 in the SI^37^. Details of the experimental routes to apply strain can be found in Section S4 of the SI^37^.

**Figure 3:**
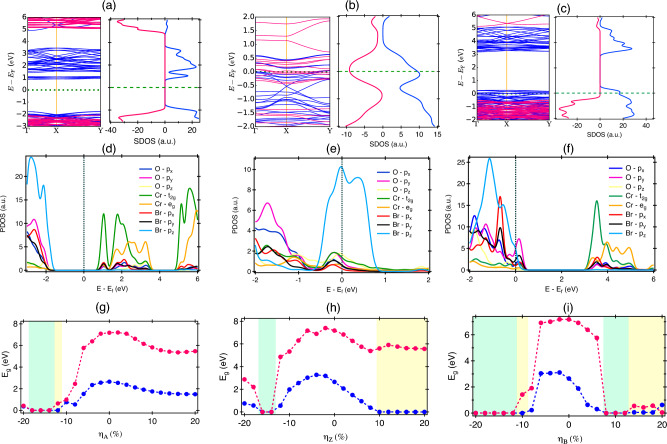
The spin-polarised band diagram (left) density of states (right) in the: (**a**) Unstrained configuration, (**b**) $\eta _{A}$ = − 16%, (**c**) $\eta _{Z}$ = 16%. Where pink and blue lines explain the contribution of spin-$\downarrow$ and spin-$\uparrow$ carriers. The projected density of states (PDOS) in the: (**d**) Unstrained configuration, (**e**) $\eta _{A}$ = − 16%, (**f**) $\eta _{Z}$ = − 16%. The color-map represents the strain induced band gap with a strain variable of: (**g**) $\eta _{A}$, (**h**) $\eta _{Z}$, (**i**) $\eta _{B}$. E$_{F}$ is the Fermi energy level.

### Electronic behaviour

#### Unstrained case

**Figure 4:**
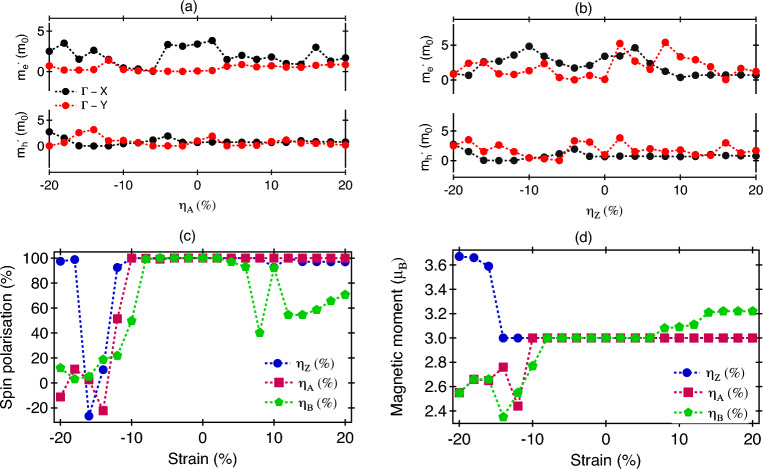
The effective mass of electron and hole with strain axis along (**a**) armchair, (**b**) zigzag direction. (**c**) Spin polarisation, (**d**) Magnetic moment of Cr atom in different strain configurations.

The electronic band structure along the high symmetry points of the monolayer CrOBr is illustrated in Fig. 3a. The spin-polarized band structures obtained from the PBE+U method demonstrated that CrOBr monolayer is a direct band gap semiconductor with an energy gap for the spin-$\uparrow$ and spin-$\downarrow$ states to be 2.647 eV and 7.177 eV respectively (Fig. 3a). The same can be confirmed from the density of states profile, where the spin-$\uparrow$ channel is semiconducting and spin-$\downarrow$ channel is insulating in nature. The minima of the conduction band is primarily contributed by the Cr-3*d* states while the hybridization of O-2*p* and Br-4*p* states are responsible for the maxima of the valence band (Fig. 3d). The results obtained using HSE06 functional can be found in Section S3 of the SI^37^. In addition, the effective mass (m*) plays an important role in the tunnelling current and in electron-hole dynamics. The m* has a complicated dependence on the crystallographic directions depending on the parabolic and non-parabolic states on the band structure, which is defined as:

8 $$\begin{aligned} \frac{1}{m^{*}_{ij}} = \frac{1}{\hbar ^{2}} \frac{\partial ^{2}E}{\partial k_{i}\partial k_{j}} \end{aligned}$$

where $\hbar$ is the reduced Planck’s constant, E is the total energy, and *k* is a wave vector. In the unstrained condition, the effective mass of electron and hole (in units of free electrons m$_{0}$) along the $\Gamma$ $\rightarrow$ X direction are estimated at 3.396 m$_{0}$, 0.68 m$_{0}$, while along the $\Gamma$ $\rightarrow$ Y direction with values of 0.076 m$_{0}$, 0.98 m$_{0}$ respectively (Fig. 4a,b). The difference in effective mass of electron indicates the anisotropic nature of the material and carriers are more mobile along the zigzag direction, which was estimated around the conduction band minimum and valance band maximum.

The magnetic ground state of monolayer CrOBr shows a large magnetic moment of 3.0 $\mu _{B}$/Cr-atom. The origin of magnetic moment in CrOBr can be related to the presence of the trivalent Cr ion (Cr$^{+3}$) in the high-spin state Cr-3*d*$^{\uparrow \uparrow \uparrow }$^27^ (Fig. 3d), while the total magnetic moment overwhelmingly stems from Cr$^{+3}$ with slight contribution from the p states of O and Br. The octahedral crystal field makes the trivalent Cr$^{+3}$ ion spilt into the half filled triply degenerated t$_{2g}$ (d$_{xy}$, d$_{xz}$, d$_{yz}$) states and empty double degenerate e$_{g}$ (d$_{x^{2}-y^{2}}$, d$_{z^{2}}$) state. The spin configuration can be depicted as t$_{2g}^{\uparrow \uparrow \uparrow }$e$^{0}_{g}$ electron.

#### Strain effect

Strain can significantly change the electronic structure of 2D materials and thus increase or decrease the band gap. Sample band diagrams were shown in Fig. 3, for specific strain cases at $\eta _{A}$ = − 16% (Fig. 3b) and $\eta _{Z}$ = 16% (Fig. 3c). The drastic modulation of energy bands in the band diagram was observed under the application of strain. The magnetic semiconductor behaviour of CrOBr changes to Half metal ($\eta _{Z}$ = 16%) and magnetic metal ($\eta _{A}$ = − 16%), which is also confirmed from the SDOS. It is observed that at $\eta _{Z}$ = 16%, spin states of spin-$\uparrow$ carriers are available at the Fermi level with high contribution of O-*p*$_{y}$ and Br-*p*$_{z}$ orbitals (Fig. 3f), while the energy gap for spin-$\downarrow$ carriers is observed to be 7.2 eV. In the $\eta _{A}$ = 16% configuration, the unequal number of density of states are available at the Fermi level for both spin types $\uparrow$ and $\downarrow$, arising from hybridization of Br-*p*$_{z}$, Cr-*t*$_{2g}$ and O-*p*$_{z}$ (Fig. 3e), which proves the magnetic metallic behaviour.

The strain-induced electronic phase transition of CrOBr along the armchair, and zigzag direction is illustrated in Fig. 3g and h. The cyan color represents the magnetic metallic behaviour, whereas the yellow color indicates the half metallic state. The band gap has sharply decreased by the application of compressive strain along the armchair direction. At, $\eta _{A}$ = − 12%, the magnetic semiconductor behaviour gets transitioned to half metallic phase and at − 12% $> \eta _{A}>$ − 20%, it revealed the presence of magnetic metal state. The band gap for the spin-$\uparrow$ states in the TS region of $\eta _{Z}$ ($\eta _{A}$ = 0%) is reducing; after $\eta _{Z}$ = 10% $\rightarrow$ 20%, the band gap is 0 eV, but the band gap for the spin-$\downarrow$ states ranges from 5.378 to 7.177 eV. The material goes through a transition from magnetic semiconductor (0% $< \eta _{Z}<$ 10%) to half-metallic (10% $< \eta _{Z}<$ 20%) state. Within the CS regime along the zigzag direction, the band gap first decreases from $\eta _{Z}$ = 0% to − 14%, and it becomes a magnetic metal at $\eta _{Z}$ = − 14%, − 16%. Above it, it becomes a magnetic semiconductor for higher values of strain. Overall, it suggests a strain induced phase transition from magnetic semiconductor $\rightarrow$ magnetic metal $\rightarrow$ magnetic semiconductor phase.

For the BS situation also, a similar observation of phase transition was observed with the presence of magnetic metal phase at $\eta _{B} \le$ -12% and 8% $\le \eta _{B} \le$ 12% as given in Fig. 3i. We observed the HM phase from 14 to 20%, where the majority of spin carriers are spin-$\uparrow$ states in comparison to spin-$\downarrow$ from 14 to 18% at the Fermi level. The switching of majority spin carriers from spin-$\uparrow$ to spin-$\downarrow$ states is observed with the increase of $\eta _{B}$ from 18 to 20%. We have also designed various distinct spin arrangements of Ferromagnetic (FM) and anti-ferromagnetic (AFM) states, as shown in Fig. S5^37^. The energy difference ($\Delta$E) within FM and AFM orientations defines the solidity of the magnetic floor, which is illustrated as $\Delta$E = E$_{AFM}$ − E$_{FM}$. The E$_{FM}$ and E$_{AFM}$ are the total energy of FM and AFM arrangements. Here, the findings reveal that the positive value of $\Delta$E makes the FM ordering more promising in comparison to AFM ordering, which is observed throughout the $\eta _{A}$ direction (Fig. S6^37^). The FM to AFM transition was observed to increase with the compressive strain by more than − 10% along the zigzag direction. In the biaxial case, we found the AFM state at − 14%, − 16%, − 18%, 8%, 10%, and 12% strain, respectively.

The application of strain changes the energy band structure of CrOBr allowing the variation in effective mass along armchair and zigzag directions along the path $\Gamma \rightarrow$ X and $\Gamma \rightarrow$ Y (Fig. 4a,b). The effective mass of an electron in the presence of an $\eta _{A}$ along $\Gamma \rightarrow$ X is lighter than the same along $\Gamma \rightarrow$ Y, inferring that an electron can transfer more quickly along $\Gamma \rightarrow$ X than along $\Gamma \rightarrow$ Y. The effective mass of the hole is equal and constant along $\Gamma \rightarrow$ X and $\Gamma \rightarrow$ Y with increasing tensile strain and lighter than the electrons. But in the CS regime, the lightest hole (0.08 m$_{0}$) was observed at $\eta _A$ = − 14%. In the $\eta _{Z}$ case, the effective mass of the hole stays constant in $\Gamma \rightarrow$ X direction and lighter than $\Gamma \rightarrow$ Y direction. In the CS case, the effective mass of hole is varying between 0.032 m$_{0}$ to 3.34 m$_{0}$ with increasing compressive strain. The lightest hole is observed at − 6% strain, while the lightest electron with effective mass of 0.06 m$_{0}$ is observed at 16% strain along $\Gamma \rightarrow$ Y direction.

Strain-dependent magnetic behavior of CrOBr can be understood from the investigation of spin polarization and magnetic moment. The Spin polarisation is measured at the Fermi level and illustrated for different strain values in Fig. 4c. Between − 10 and 6%, the system shows high spin polarisation of 100% independent of the nature of applied strain. For $\eta _{A}$, perfect spin filtering is observed between − 10 and 20% strain, while for $\eta _{Z}$, it is − 10% to 8% and in the presence of $\eta _{B}$ from − 8 to 2%. For most part of the strain range, the system shows perfect spin polarisation originating from the dominance of one type of spin states and large presence of half metallicity in CrOBr. Some departure happens at larger values of applied strain both in the CS and TS scenario. A large value of spin polarisation can be useful for using CrOBr as a spin-injector or spin filtering in a spin circuit. The magnetic moment (Fig. 4d) have a maximum of 3.67 $\mu _{B}$ at $\eta _{Z}$ = − 20% strain, minima of 3 $\mu _{B}$. For most part of strain, moment stays at 3 $\mu _{B}$. For $\eta _{B}$, it increases to 3.22 $\mu _{B}$ at strain > 14 %, while for CS along $\eta _{A}$ and $\eta _{B}$, it shows a reduction in moment to a value of 2.35 $\mu _{B}$. The value of moment is comparable to that of CrOCl (~3 $\mu _{B}$/Cr atom^17^), CrI$_{3}$ (~3 $\mu _{B}$/Cr atom^38^) and other materials [CrOF (~3.06 $\mu _{B}$/Cr atom^39^), CrCl$_{3}$ (~3.08 $\mu _{B}$/Cr atom^40^)]. A large value of magnetic moment suggests a stronger presence of the magnetic ordering and high internal field associateed with it.

**Figure 5:**
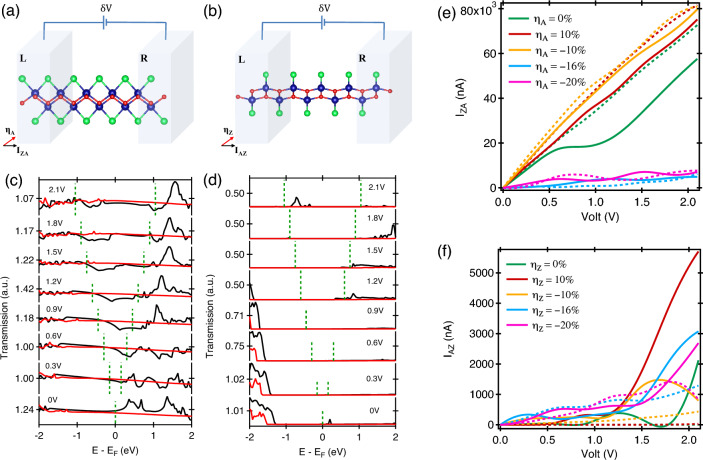
The two-probe geometry of monolayer CrOBr with measuring current along (**a**) zigzag direction under $\eta _{A}$ scenario, (**b**) armchair direction with $\eta _{Z}$ variation. The transmission probability under unstrained configuration with external bias along (**c**) zigzag, (**d**) armchair direction. The black color represents the spin-$\uparrow$ state and red color is for the spin-$\downarrow$ state. The I–V characteristics of measuring current along: (**e**) zigzag, (**f**) armchair direction. The solid lines are using for spin-$\uparrow$ current (I$_{\uparrow }$) and dotted lines are for spin-$\downarrow$ current (I$_{\downarrow }$).

**Figure 6:**
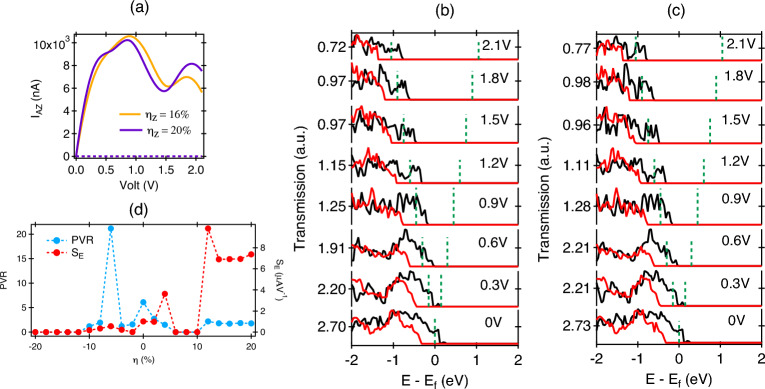
(**a**) The I$_{AZ}$-V characteristics of measuring current along armchair direction. The transmission probability of: (**b**) $\eta _{Z}$ = 16%, (**c**) $\eta _{Z}$ = 20%. (**d**) The PVR and S$_{E}$ curves are drawn as function of strain for spin $\uparrow$ current along zigzag direction.

**Figure 7:**
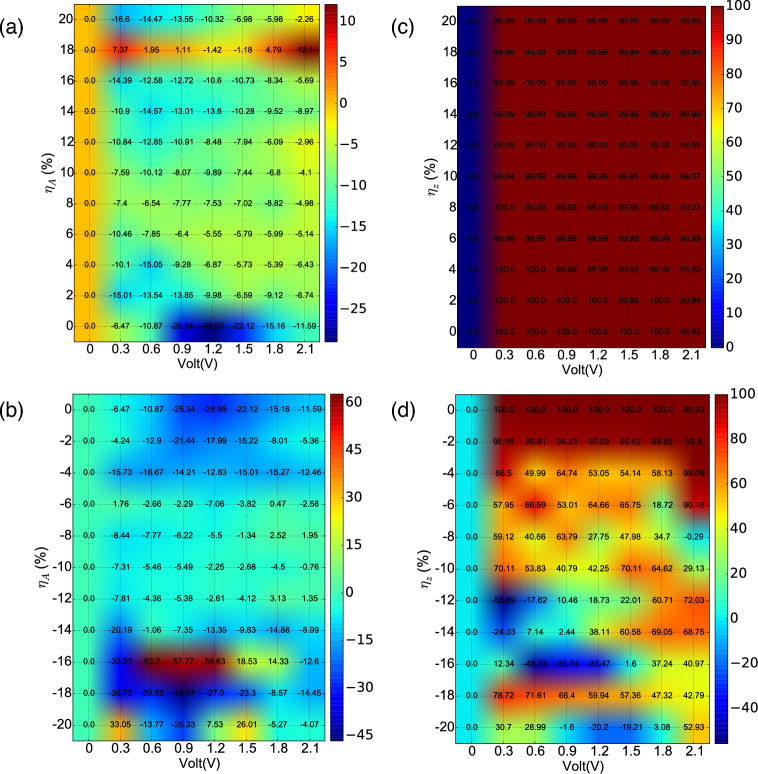
The phase diagram of SIE with bias voltage under strain application of: (**a**, **c**) $\eta _{A}$, (**b**, **d**) $\eta _{Z}$.

### Transport behaviour

We measured the transport behavior employing a two-probe geometry, coupled with a scattering region connected between left (L) and right (R) electrodes as shown in Fig. 5a and b. The current–voltage (IV) characteristics of monolayer CrOBr was investigated with an uniaxial strain applied in two perpendicular directions to the transport axis: (i) measuring current along the zigzag direction, where the strain was applied along the armchair axis, I$_{ZA}$ (Fig. 5e). (ii) current measured along the armchair direction for strain applied along zigzag direction, I$_{AZ}$ (Fig. 5f). Further, the current was split into two spin-specific components: spin-up (I$_{ZA\uparrow }$ and I$_{AZ\uparrow }$) and spin-down (I$_{ZA\downarrow }$ and I$_{AZ\downarrow }$). The conduction behaviour of the CrOBr monolayer for different strain values is displayed in Fig. 5e and f.

In the presence of $\eta _{A}$ in Fig. 5e, at $\eta _A = 0\%$, no current flows at zero bias, but it starts to increase linearly with an increase in applied voltage from 0 V $\rightarrow$ 2.1 V. There is a majority of spin-$\downarrow$ current in total, which shows ohmic behavior. In the spin-$\uparrow$ case, the current is raised linearly from 0 to 0.7 V, and stays constant for the interval between 0.7 $\rightarrow$ 0.9 V, after which I$_{ZA}$ exhibits an ohmic response. This can be confirmed from the transmission behaviour as illustrated in Fig. 5c at different bias voltages. The transmission probability of spin-$\downarrow$ is higher than spin-$\uparrow$ carriers. With an increase in the CS, the nature of the IV response stays Ohmic for $\eta _{A}$ = -10% while the value of current gets reduced drastically for $\eta _{A}$ = -16% and above. Overall, the spin-$\downarrow$ is found to be the majority type here. The transmission spectrum at different values of $\eta _{A}$ are shown in Fig. S1 of supporting information (SI)^37^. In Fig. 5f, the IV pattern shows interesting conduction oscillations for different values of $\eta _{Z}$, details of which is explained later. Here, at low voltages, both the spin types contribute significantly in conduction but spin-$\uparrow$ becomes the majority type at higher voltages (Fig. 5d,f). The maximum current is observed at $\eta _{Z}$ = 10% at 2 V with a value of 5705 nA. Comparing the conduction along armchair and zigzag direction, it can be said that overall a higher level for conduction is observed along zigzag direction compared to the zigzag path. Interesting conduction behaviour was observed at $\eta _{Z}$ = 16% (see Fig. 6a) where the current in the Spin-$\uparrow$ channel is first found to increase rapidly with an increase in the applied bias with a maximum occurring at 0.9 V, beyond which it starts to decrease and increase again above 1.5 V. Between 0.9 V $\rightarrow$ 1.5 V, the current decreases while the voltage increases, resulting in a negative differential resistance called as NDR effect. Similar effect is also observed at $\eta _{Z}$ = 20%, which can be explained further using the transmission behaviour given in Fig. 6b and c. Between 0 and 2.1 V, the transmission in the spin-$\uparrow$ channel increases within the applied bias window marked by the dotted lines, which gets reduced between 0.9 and 1.5 V and further increases beyond that. The current value is dependent on the integrated sum of the transmission within the marked voltage range. Similar observation can be made for the transmission given in Fig. 6d for $\eta _{Z}$ = 20%. It is to be noted that in both cases, spin-$\downarrow$ channel remains unaffected from such oscillations. Further quantification in NRD can be made via PVR, S$_{E}$ given by,

9 $$\begin{aligned} \hbox {PVR}= & {} \ \mid \frac{\hbox {I}_{\textrm{peak}}}{\hbox {I}_{\textrm{valley}}}\mid \end{aligned}$$

10 $$\begin{aligned} \hbox {S}_{\textrm{E}}= & {} \frac{\mid \hbox {I}_{\textrm{peak}} - \hbox {I}_{\textrm{valley}} \mid }{\mid \hbox {V}_{\textrm{peak}} - \hbox {V}_{\textrm{valley}} \mid } \end{aligned}$$

where, the current and voltage at the peak and valley positions of the I-V characteristics are represented by the symbols (I$_{peak}$, V$_{peak}$) and (I$_{valley}$, V$_{valley}$), as shown in Fig. S3 of SI^37^. PVR (peak-to-valley ratio) is a quantification of the current limit through which an ON/OFF operation is carried out, while S$_{E}$ (switching efficiency) measures how quickly a system can be shifted between a peak and a valley location. When there is no strain $\eta _{Z}$ = 0%, the observed PVR of 6.105 with S$_{E}$ is 1.009 $\mu$AV$^{-1}$ for spin-$\uparrow$ carriers (Fig. 6d). The highest PVR observed is 2.724 with S$_{E}$ = 9.8237 $\mu$AV$^{-1}$ at $\eta _{Z}$ = 2%. In the CS region, the maximum PVR of 21.162 with S$_{E}$ is 0.558 $\mu$AV$^{-1}$ at $\eta _{Z}$ = − 6%.

The majority contribution in total current between two types of spin carriers are measured through spin injection efficiency (SIE), which is defined as:

11 $$\begin{aligned} \hbox {SIE} = \frac{\hbox {I}_{\uparrow } - \hbox {I}_{\downarrow }}{\hbox {I}_{\uparrow } + \hbox {I}_{\downarrow }} \times 100\% \end{aligned}$$

The spin injection behaviour as a function of applied voltage for different strain cases is shown in Fig. 7. In Fig. 7a, it can be observed that the highest SIE has a value of -28.95% in the unstrained condition, which stays of the same order of magnitude with an increase in the applied bias. A negative value of SIE indicates the Spin-$\downarrow$ states to be majority carrier type, which is true for most part of the phase diagram, except at $\eta _{A}$ = 18%, where spin-$\uparrow$ is the majority type with a maximum value of SIE of 12.1%. The low value of SIE in Fig. 7a can be related to the significant spin transmission occurring along both the spin channels in the TS scenario. However, the SIE improves significantly in the CS case applied along the armchair direction as shown in Fig. 7b. The highest value of SIE of 62.7% was observed at $\eta _{A}$ = − 16% at 0.6 V, while the SIE goes through a change of sign at this strain value with a change of applied bias. The change of the nature of majority spin character can be routed through the oscillating nature of I-V characteristics at this strain value. For other values of $\eta _{A}$, the SIE stays negative with noticeable values present between $\eta _{A}$ = 0 to − 4% and − 16 to − 20%. However, drastic change in the SIE character is observed in the presence of $\eta _{Z}$, where almost perfect spin injection is found in the TS regime as illustrated in Fig. 7c. Here spin-$\uparrow$ is the majority type and the origin of the high value of SIE can be linked to presence of spin-specific NDR effect and half-metallic nature of the material in this strain range. Here one of the two spin channels is conducting highly, the contribution from the other spin channel is negligible. In the CS region (Fig. 7d), the SIE is significantly high for low values of strain and decreases with an increase in $\eta _{Z}$. A change of sign in SIE is detected for intermediate and high strain values. Overall, it can be observed that the system shows perfect spin filtering behaviour for large part of the phase diagram, which is further tunable with applied bias and strain to reach desired value and sign. Such type of strain tunable SIE response could have versatile application in quantum spintronics and straintronics.

## Conclusion

In summary, we have examined the spin-splitted electronic band structure of 2D-CrOBr under strain engineering and the spin-dependent transport properties in various device geometry. The results indicate that the band structure of monolayer CrOBr is spin distinguished with different magneto-electric phases which can be achieved by applying uniaxial and biaxial strains. Starting from magnetic semiconductor state, to half-metallic behaviour to a magnetic metal, CrOBr shows rich phase tunability using strain variation. This leads to spin -resolved conductance and IV characteristics showing negative differential conductance behaviour. Especially, a perfect spin-filtering effect arises with a high spin injection efficiency $\sim$ 100% for transport occurring along armchair direction. The corresponding mechanisms are analyzed by the band structure with the spin resolved electron transmission spectra. These results could trigger significant interest in exploration of strain engineered efficient magneto-electronic and spintronic devices.

### Supplementary Information

Supplementary Information.

## Data Availability

The data that support the findings of this study are available from the corresponding author(s) upon reasonable request.
