# Supplementary material for: Strain-controlled spin transport in a two-dimensional (2D) nanomagnet
Source: Sci Rep. 2023 Oct 3;13:16599. doi: 10.1038/s41598-023-43025-w (PMC10547692; doi:10.1038/s41598-023-43025-w)
Supplement: Supplementary file 1 — Supplementary Information. [file 41598_2023_43025_MOESM1_ESM.pdf]

Supporting Information

Strain-controlled spin transport in two-dimensional (2D) nanomagnet

P. Kumari[a], S. Rani[a], S. Kar[a], M. Venkata Kamalakar[b], S. J. Ray[a,1]

[a]Department of Physics, Indian Institute of Technology Patna, Bihta 801106, India

[b]Department of Physics and Astronomy, Uppsala University, Box 516, SE-75120 Uppsala, Sweden

[1] Email: ray@iitp.ac.in, ray.sjr@gmail.com

S1: Transmission spectrum at different values of η_A

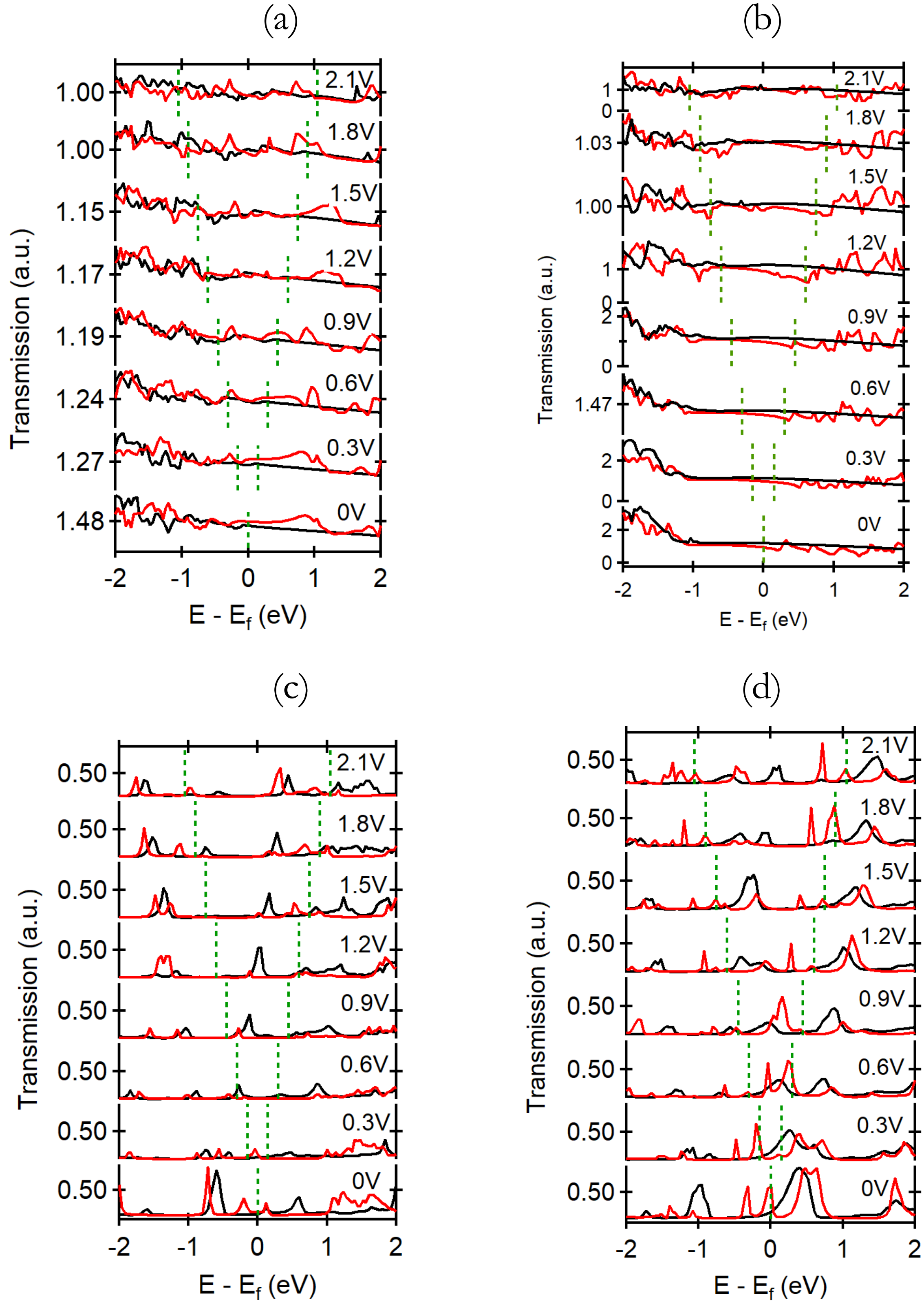

Figure S1: The transmission spectrum of CrOBr at (a) η_A = 10%, (b) η_A = -10%, (c) η_A = -16%, (c) η_A = -20% corresponding to I_{ZA}-V.

S2: Transmission spectrum at different values of η_Z

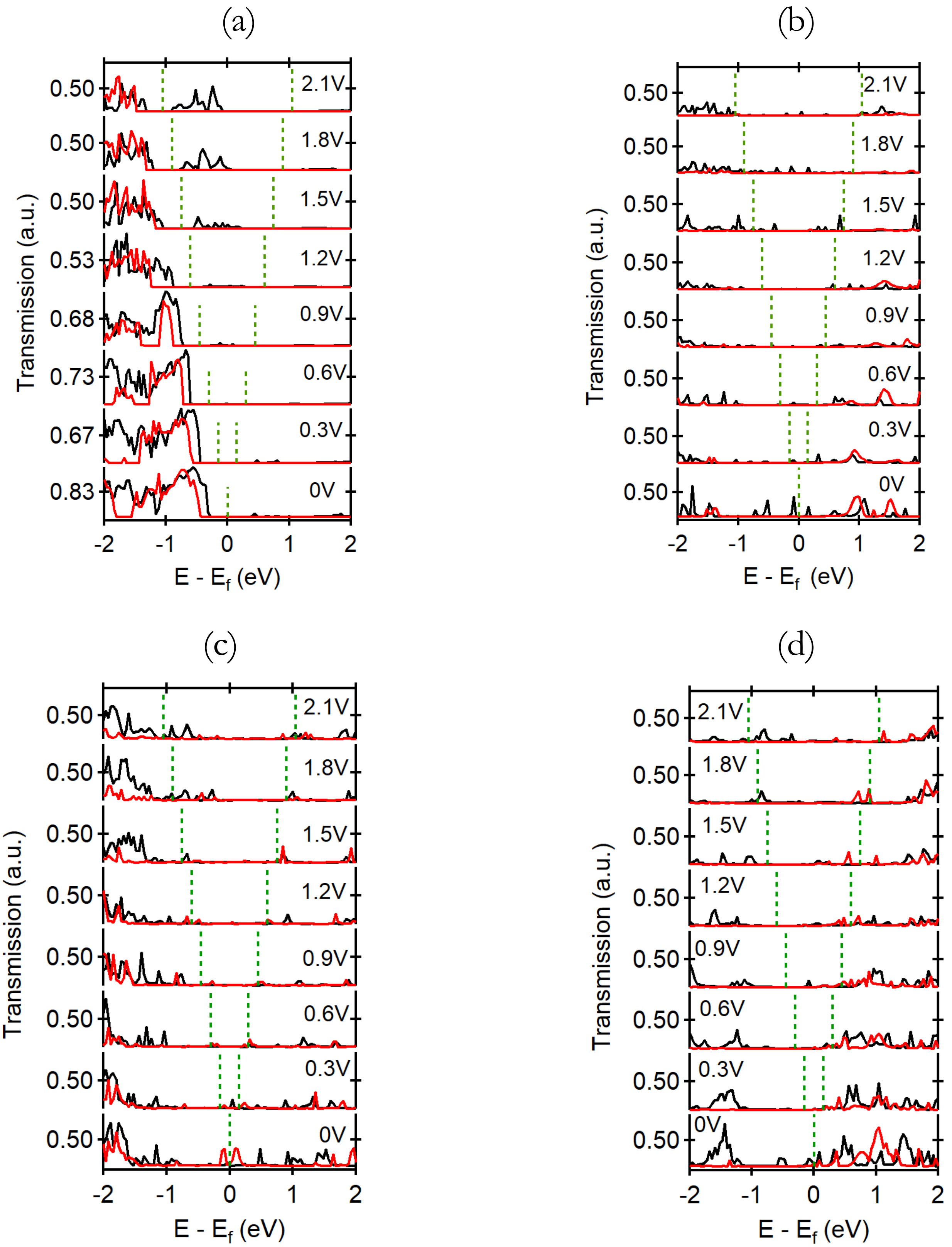

Figure S2: The transmission spectrum of CrOBr at (a) η_Z = 10%, (b) η_Z = -10%, (c) η_Z = -16%, (c) η_Z = -20% corresponding to I_{AZ}-V.

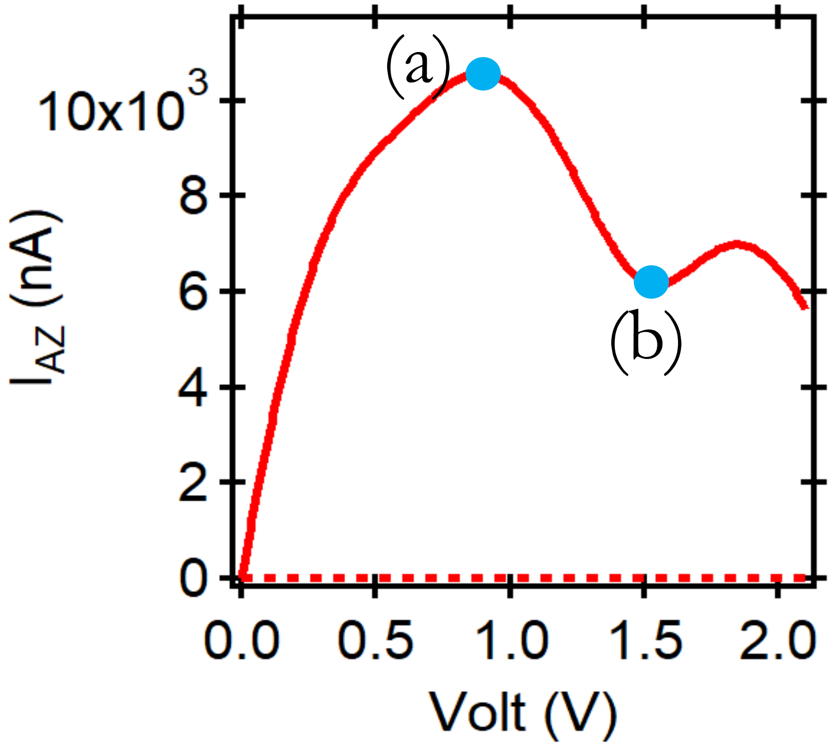

Figure S3: The I_{AZ}-V curve at η_Z = 16%, where the solid line represents the spin - ↑ current and the dotted line for a spin - ↓ current. The (a) position is the highest peak current I_{AZ}^{peak} and (b) is the valley current I_{AZ}^{valley}.

S3: The phonon band structure along η_A direction:

We have addressed the stability, to begin by comparing the bond lengths and bond angles of the optimized geometries under the compressive strained (η_A > -10%) and unstrained CrOBr, which is listed in Table R1. We do not observe any large deviation of bond lengths and bond angles between unstrained and strained conditions. Additionally, a comparison of the total energy from unstrained to strained configurations is also shown in Figure 2c in the main manuscript; which starts to decrease after η_A exceeds -16%. We also described the dynamic stability of the strained structure through the phonon band structure (Figure R1). There is no presence of negative frequency in compressive strained configuration, supporting the mechanical reliability of the structure.

Table S1: The bond length and bond angle are listed of the optimized geometries under the compressive strained (η_A > -10%)

η_A (%)	Cr – O (Å)	Cr – Br (Å)	O – Cr - O	Br – Cr – Br
-12	1.79	2.51	150.08°	83.30°
-14	1.93	2.54	143.69°	80.02°
-16	2.03	2.57	147.23°	85.99°
-18	2.12	2.61	160.54°	84.68°
-20	1.95	2.57	115.33°	80.70°
Unstrained CrOBr	1.9912	2.48683	154.20°	86.46°

(a)

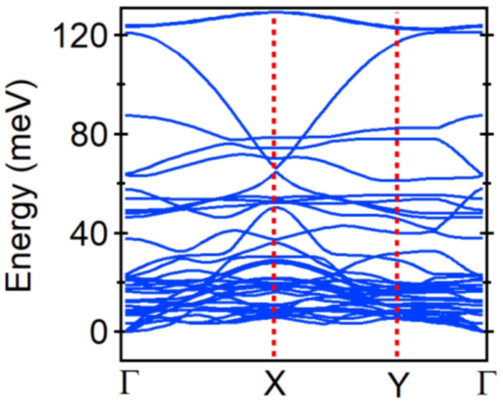

(b)

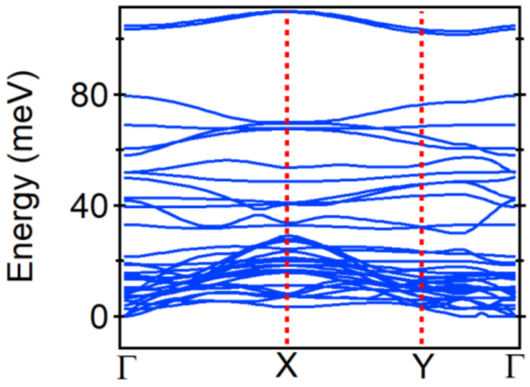

(c)

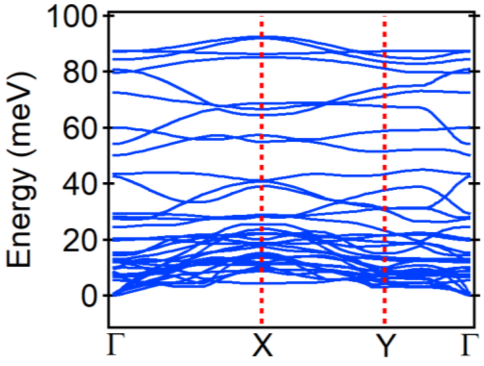

(d)

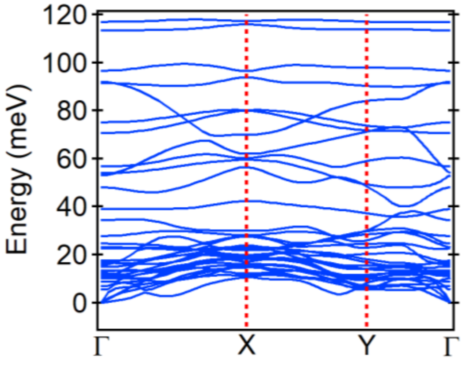

(e)

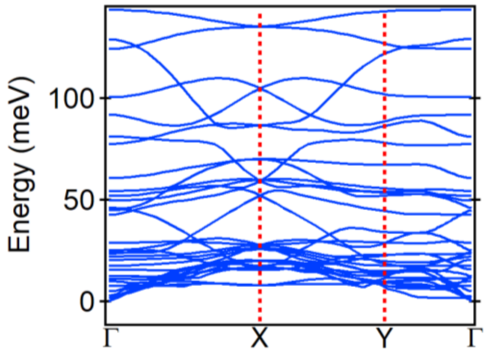

Figure S4: The phonon band structure for η_A of: (a) -12%, (b) -14%, (c)-16%, (d)-18%, and (e)-20%. The supercell size of 3 × 1 × 1.

S3: FM-AFM phase transition:

We have investigated the FM-AFM phase transition under various strain engineering. We have designed distinct spin arrangements in FM and AFM states, as shown in Figure R2. The energy difference (ΔE) within FM and AFM orientations defines the solidity of the magnetic floor, which is illustrated as $\Delta E = E_{AFM} - E_{FM}$. The E_{FM} and E_{AFM} are the total energy of FM and AFM arrangements. Here, the findings reveal that the positive value of ΔE makes the FM ordering more promising in comparison to AFM ordering, which is observed throughout the η_A direction (Figure R3). The FM to AFM transition was observed to increase with compressive strain when it goes above -10% along the zigzag direction. In the biaxial case, we found the AFM state at -14%, -16%, -18%, 8%, 10%, and 12% strain values, respectively.

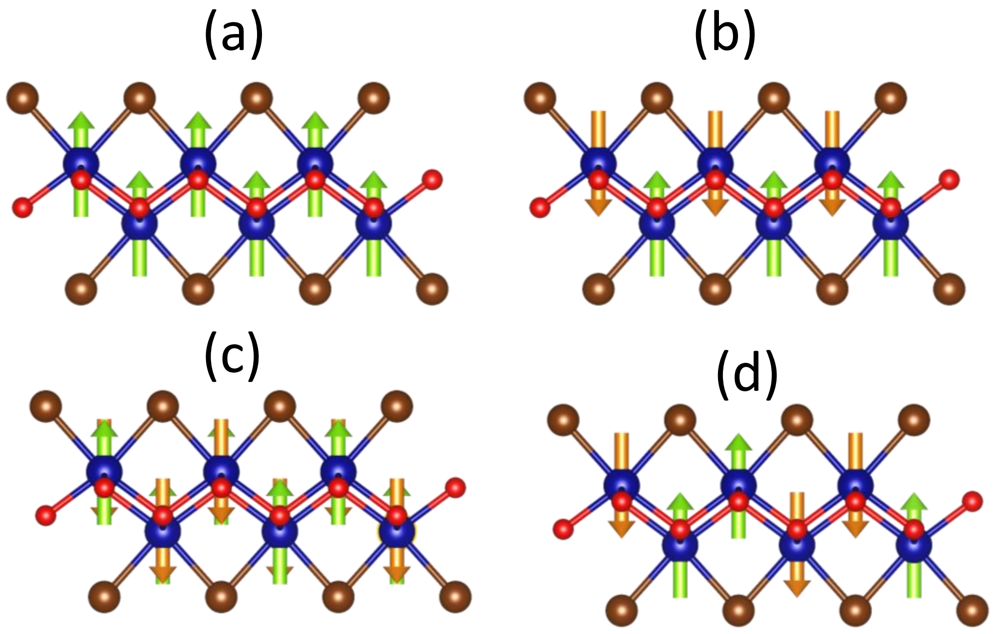

Figure S5: The various FM and AFM configuration of 2D CrOBr: (a)FM, (b)AFM-1, (c)AFM-2, (d)AFM-3.

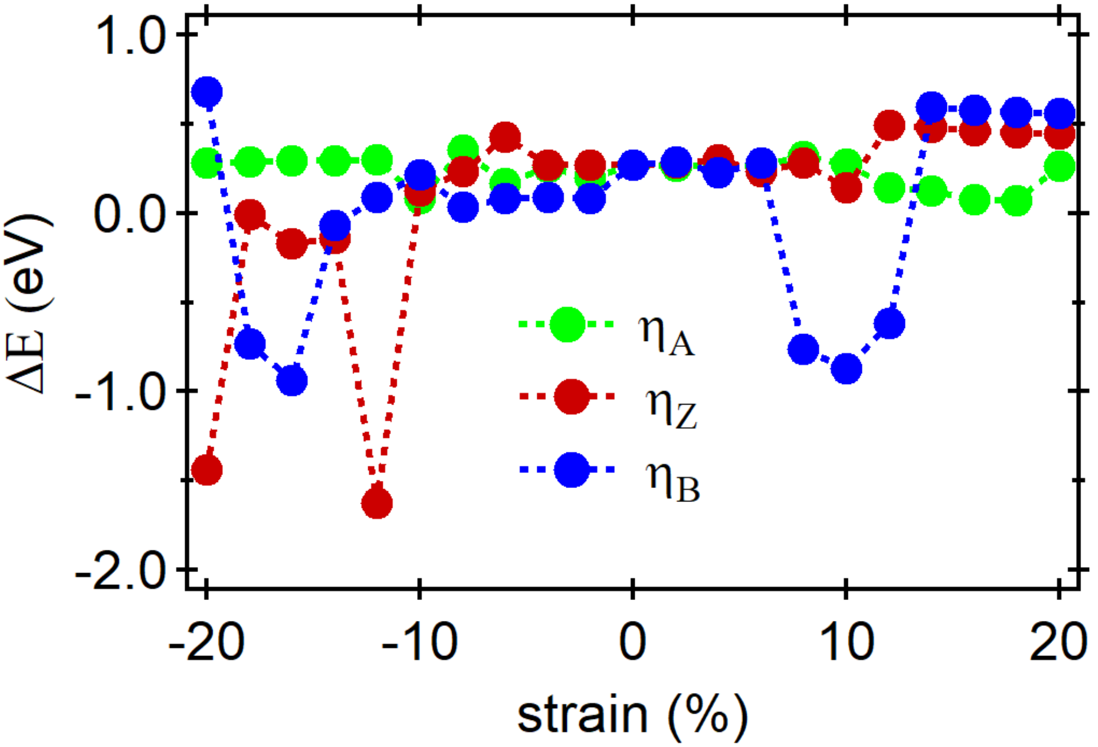

Figure S6: The energy difference between FM and AFM configuration.

S3: The band structure through HSE and GGA+U calculation

The electronic properties computed through DFT are dependent on the exchange-correlation functional and the hybrid functionals are computationally heavily demanding. For a range of 2D materials including recently discovered 2D layered magnets, GGA and GGA+U have been used extensively **(Nanoscale Horizon, 2018, 3, 335, Nanoscale, 2018,10, 14298-14303, Chem. Mater. 2015, 27, 612−620, ACS Nano, 6, 1695–1701, 2012, J. Mater. Chem. C, 2017, 5, 8734, Phys. Chem. Chem. Phys., 2016,18, 8777-8784, Journal of Applied Physics 117, 064313, 2015)** etc. with reliable predictions, which justifies the use of the current methodology for the present work. It is to be noted that, the results obtained through Hybrid functional like Heyd−Scuseria−Ernzerhof (HSE06) were found to be highly consistent with the DFT-PBE (GGA) based calculations while investigating the strain effects on the electronic band structures, the direct-indirect band gap transition and the gap variation trends with strain **(Physical Rev. B 90, 085402, 2014, Physical Review Applied 11, 024056, 2019, Physical Chemistry Chemical Physics 20, 13508-13516, 2018)** for various 2D systems. It validates the current calculation methodology used for CrOBr.

The sample band structure of 2D CrOBr is shown below (Figure S7), which shows semiconducting behaviour with indirect band gap for spin-$\uparrow$ and spin-$\downarrow$ states with values of 3.45 eV and 6.63 eV respectively, which are higher than the PBE + U calculation for spin-$\uparrow$ electrons and lower for spin-$\downarrow$. We have also calculated the band structure at η_z = -14%, 12%. We observed the metallic and half-metallic behaviour like the PBE+U method. At η_z = 12%, the spin-$\uparrow$ band gap of 2.673eV is lower than the PBE+U calculation. Whereas, the spin-$\downarrow$ band gap is 0 eV. It was observed that although the exact band gap values are different in both methods, but the overall trend in its variation observed with strain remains similar, hence strengthening our findings.

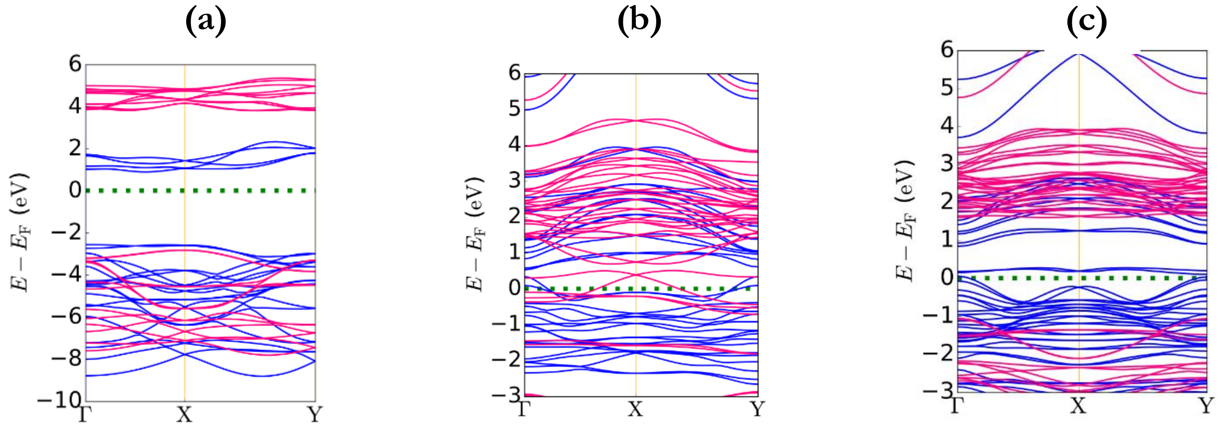

Figure S7: The band structure of 2D CrOBr through HSE calculation at: (a) unstrained condition, (c)η_Z = -14%, and (e) η_Z = 12%. The blue and pink color represent spin-↑ and spin-↓ states respectively.

S4: Experimental scope of strain-controlled studies

Popular 2D materials like graphene, MoS_2, and Phosphorene stay within the elastic limit of up to a larger strain of around 30% [Science, 2008, 321, 385–388; Nanoscale Res. Lett., 2012, 7, 233; Nano letters 16.5 (2016): 2931-2937]. Experimentally, strain can be applied through a bending apparatus as used for bending MoS_2 on a polycarbonate substrate [Nano Lett. 2013, 13, 3626−3630] or nano-indentation in an atomic force microscope [Science, 321, 385-388, 2008]. The commonly flexible substrate can also be used applying strain. However, for applying preferential strain one has to identify the edge structure (zigzag or armchair). In general, such measurements can be done by exfoliating the flakes of the 2D later over a flexible substrate and using high-resolution atomic force microscopy to understand the orientation. A very recent article [DOI: 10.1126/sciadv.aar7181] reported a similar observation with graphene nanoribbon where individual dopant atoms can be identified through the AFM tip. A monolayer film of FeSe on $SrTiO_3$(110) substrate [FeSe/STO(110)], with a C2 symmetry induced by epitaxial strain 6% [Physical Review B 94, no. 10 (2016): 104510]. Whereas, PbI_2 maintains a direct bandgap nature under a large experimental strain up to 7.69% [2D Materials 6, no. 2 (2019): 025014]. Many reports are available, which restrict to small strain values only [Nature Nanotechnology 17.3 (2022): 256-261; npj 2D Materials and Applications 6, no. 1 (2022): 41]. However, by extending the methodology to high strain rates, we were able to observe phase transition in the material, where the system swapped its current semiconductor ground state to being Half metal while completely staying in the elastic regime without any plastic deformation.
